# Nuclear endonuclease G controls cell proliferation in ovarian cancer

**DOI:** 10.1002/2211-5463.13572

**Published:** 2023-02-13

**Authors:** Ye Na Choi, Tae Woong Seo, Yui Taek Lee, Dar Heum Jeong, Soon Ji Yoo

**Affiliations:** ^1^ Department of Biology Kyung Hee University Seoul South Korea; ^2^ Department of Life and Nanopharmaceutical Sciences Kyung Hee University Seoul South Korea

**Keywords:** antioxidants, cell death, cell proliferation, endonuclease G (EndoG), ovarian cancer (OC), reactive oxygen species (ROS)

## Abstract

Ovarian cancer is characterized by a high degree of genetic heterogeneity. Platinum‐based chemotherapy and some gene‐targeted therapies have shown limited treatment efficacy due to toxicity and recurrence, and thus, it is essential to identify additional therapeutic targets based on an understanding of the pathological mechanism. Here, we report that endonuclease G, which exhibits altered expression in ovarian cancer, does not function as a cell death effector that digests chromosomal DNA in ovarian cancer. Endonuclease G is modulated by intracellular reactive oxygen species dynamics and plays a role in cell proliferation in ovarian cancer, suggesting that targeting endonuclease G alone or in combination with other antitumor agents may have the potential for development into a treatment for endonuclease G‐overexpressing cancers, including ovarian cancer.

## Graphical abstract

Endonuclease G (EndoG) is a mitochondrial endonuclease that is translocated to the nucleus during apoptosis. In ovarian cancer (OC), however, EndoG is upregulated and nuclear‐localized due to elevated reactive oxygen species (ROS) and plays a proliferative function. Decreasing ROS levels reduces OC cell proliferation and enhances cisplatin efficacy by reducing EndoG availability, suggesting targeting EndoG as a promising option for OC treatment.

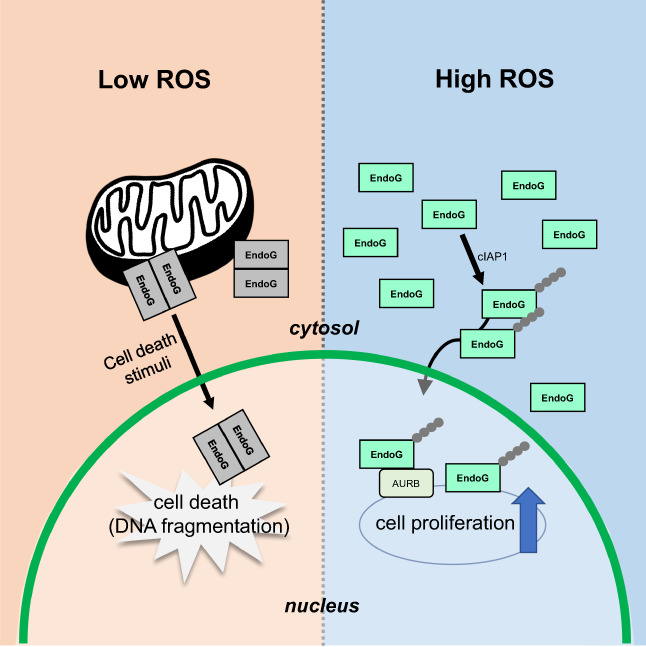

**Abbreviations**
2‐DG: 2‐deoxy‐d‐glucose
BIR: baculoviral IAP repeat
CHIP: C‐terminus of Hsc70‐interacting protein
CHX: cycloheximide
cIAP1: cellular inhibitor of apoptosis protein
CPC: chromosome passenger complex
DAPI: diamidino‐2‐phenylindole
EndoG: endonuclease G
FBS: fetal bovine serum
GAPDH: glyceraldehyde 3‐phosphate dehydrogenase
GEPIA: gene expression profiling interactive analysis
GFP: green fluorescent protein
H_2_O_2_
: hydrogen peroxide
NAC: N‐acetyl cysteine
PBS: phosphate‐buffered saline
PI: propidium iodine
qRT‐PCR: quantitative reverse‐transcription polymerase chain reaction
ROS: reactive oxygen species

Ovarian cancer (OC) is a common gynecologic malignancy. It is often diagnosed at an advanced stage due to a lack of symptoms in the early stage, and thus the 5‐year survival rate is poor [1]. Platinum‐based chemotherapy, which induces DNA instability, is the standard treatment for OC, and cisplatin is the most frequently used drug for OC treatment. However, the efficacy of cisplatin is limited due to the development of drug resistance [1, 2]. Therefore, it is important to provide additional treatment options for OC patients, including those used in combination with platinum agents or single gene‐targeted therapies. Genes associated with OC have been discovered, and targeted therapies have been developed for some genes [3, 4, 5]. Bevacizumab targets the VEGF pathway, and PARP inhibitors such as olaparib can treat certain OC subtypes [4, 5]. However, in addition to toxicity, disease relapse is quite common after primary chemotherapy [4, 5, 6]. OC patients have a particularly heterogenous genetic background [1]. Therefore, the identification of additional altered genes in OC and understanding their tumorigenic mechanism are essential to expanding treatment options.

Reactive oxygen species (ROS) are naturally generated in cells, mostly as by‐products of metabolic processes. Various forms of ROS are eventually reduced to hydrogen peroxide (H_2_O_2_). Accumulation of ROS can cause oxidative stress, which is toxic to organisms, thus the antioxidant system must maintain low ROS levels under normal physiological conditions. However, the redox system, which controls intracellular ROS levels, is frequently dysregulated in cancers [7]. According to a study of OC patients, the ROS concentration was approximately 96% higher in malignant tissues compared to that in normal tissues [8], implying that high ROS levels might cause the proliferation and survival of OC cells. Indeed, antioxidants are used for OC therapy [9, 10], but the underlying mechanism is not fully understood.

Endonuclease G (EndoG) is a cell death effector that causes DNA fragmentation during apoptosis [11, 12]. EndoG is localized in mitochondria under normal conditions. EndoG is an evolutionary conserved DNA/RNA digesting nuclease [13] and target preference between DNA and RNA is changed depending on physiological ionic strength [14]. When apoptosis is triggered, EndoG is released from mitochondria and moves to the nucleus, destroying chromosomal DNA. Various types of cells are killed when nuclear EndoG translocates from the mitochondria during oxidative stress [15, 16]. Interestingly, EndoG expression is induced by oxidative stress, including H_2_O_2_ treatment, as shown in our previous study [17]. Furthermore, elevated EndoG levels can sensitize cancer cells to chemotherapeutic drugs [18, 19]. Regarding cancer cell death, studies have collectively suggested that increasing ROS levels might be a possible strategy to destroy cancer cells via nuclear EndoG activity because oxidative stress would induce EndoG expression and translocation to the nucleus [20, 21]. However, in OC therapy, various types of antioxidants have been used to decrease intracellular ROS alone or in combination with other antitumor reagents [9, 10]. This strategy conflicts with the classical apoptotic function of oxidative stress‐induced EndoG, suggesting that EndoG might be differentially regulated by oxidative stress in OC.

Surprisingly, we found that EndoG is upregulated and localized in the nucleus in OC cell lines and patient tissues under normal conditions without additional apoptotic insult. We hypothesized that EndoG might not play an apoptotic role in OC. Therefore, we investigated whether EndoG might have a vital function in OC and whether it can be used as a therapeutic option alone and in combination with platinum‐type agents for OC.

## Results

### EndoG does not function as a cell death effector upon oxidative stress in OC cells

**Fig. 1:**
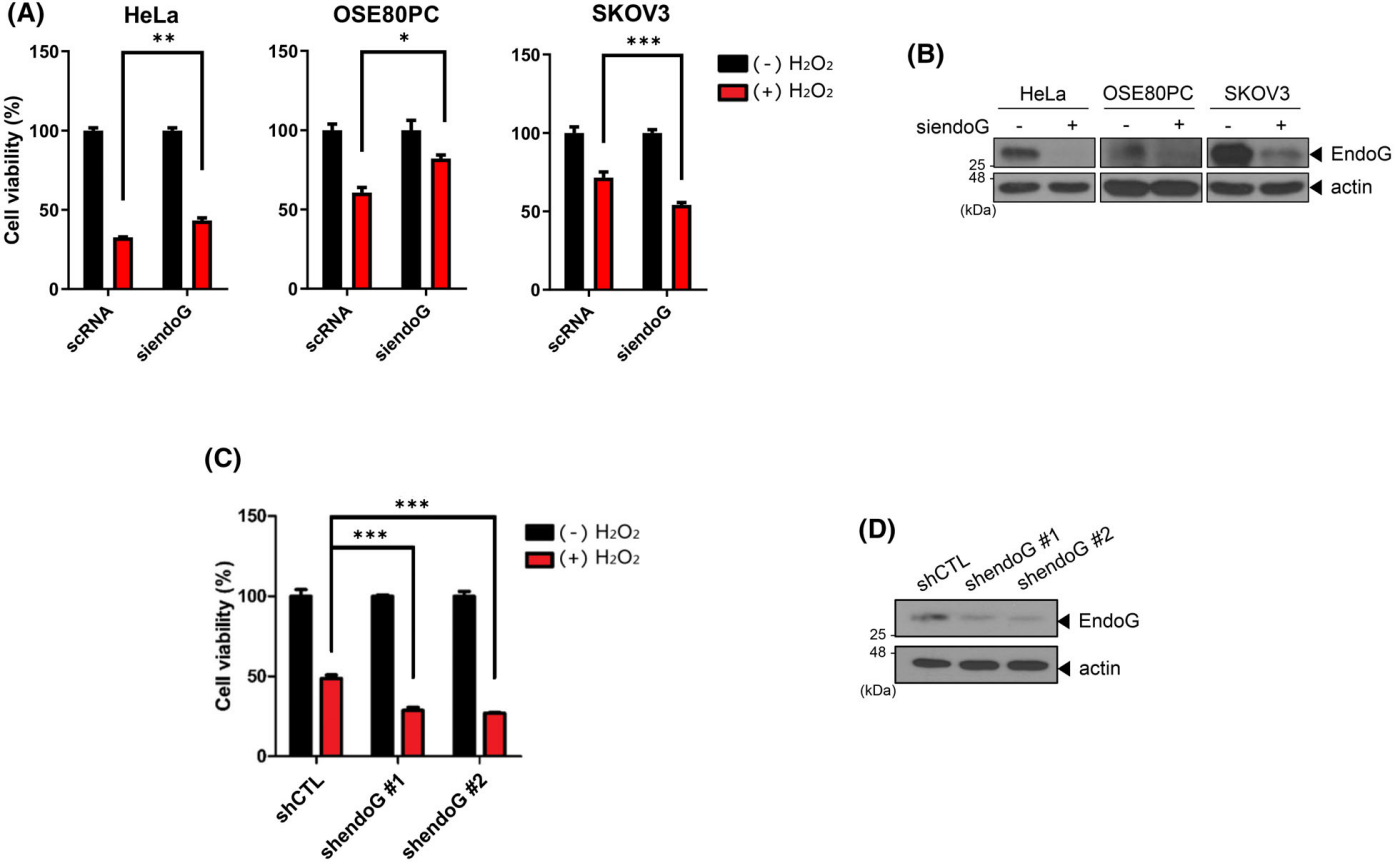
EndoG does not function as a cell death effector under oxidative stress in OC cells. (A) HeLa, OSE80PC (normal ovary cell line), and SKOV3 (OC cell line) cells were transfected with scRNA or siendoG for 48 h and treated with 500 μm (OSE80PC, SKOV3) or 1 mm (HeLa) H₂O₂ for 4 h. The cell viability was analyzed with a WST assay. (B) The RNAi efficiency in (A) was evaluated by WB with anti‐EndoG and antiactin antibodies. (C) EndoG‐KD cells (shendoG) and control cells (shCTL) were treated with 500 μm H₂O₂ for 4 h. The cell viability was analyzed with a WST assay. (D) The EndoG levels in (C) were analyzed by WB. The means ± SEM are given for three independent experiments, ***P < 0.001, **P < 0.01, *P < 0.05, unpaired *t*‐test (A, C).

Previously, we showed that EndoG is responsible for oxidative stress‐induced cell death in HeLa cells and primary rat cortical neurons [17]. EndoG acts as a death effector and cleaves chromosomal DNA upon H_2_O_2_ treatment [11, 12]. While exploring a novel function of EndoG in various cancer types, we noticed that EndoG might not be involved in cell death in OC cells. A water‐soluble tetrazolium (WST) assay showed decreased cell viability of EndoG‐depleted SKOV3 cells (an OC cell line) treated with siendoG and exposed to oxidative stress and increased viability in EndoG‐depleted OSE80PC (control normal ovary cell line) and EndoG‐depleted HeLa cells (Fig. 1A,B), consistent with our previous results [17]. The established stable cell line in this study, SKOV3 cells with EndoG‐knockdown via shendoG (EndoG‐KD cells), again showed decreased cell viability upon oxidative stress compared to control cells (shCTL cells) (Fig. 1C,D). If EndoG were a cell death effector upon oxidative stress in OC, EndoG‐depleted cells would have shown increased cell viability. These results indicate that in OC cell lines, EndoG is not a cell death effector in response to oxidative stress and may have a nonapoptotic function.

### Upregulated EndoG is localized in the nucleus in OC cells

**Fig. 2:**
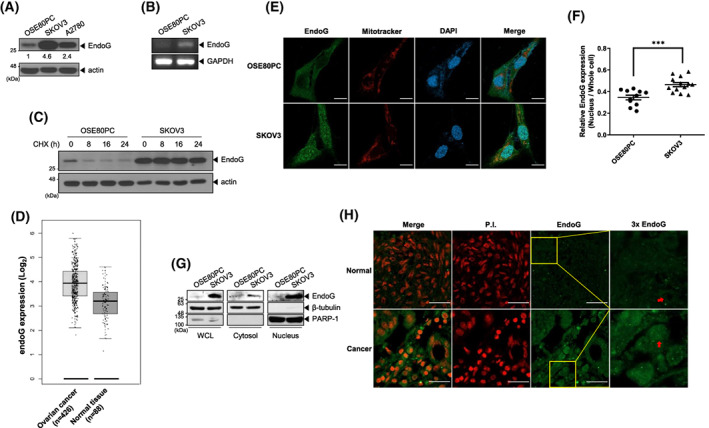
Upregulated EndoG is localized in the nucleus and cytosol in OC cells. (A) The steady‐state levels of EndoG were analyzed by WB with an anti‐EndoG antibody in OSE80PC cells and the SKOV3 and A2780 OC cell lines. The numbers indicate the expression of EndoG relative to that of Actin. (B) mRNA expression of *endoG* via RT‐PCR in normal ovary cells (OSE80PC) or OC cells (SKOV3). GAPDH was used as an internal control for RT‐PCR. (C) OSE80PC and SKOV3 cells were treated with 200 μg·mL^−1^ CHX and harvested at the indicated times. WCLs were analyzed by WB. (D) The *endoG* mRNA expression was analyzed using the GEPIA database (http://gepia2.cancer‐pku.cn). The box plots of 426 OC samples (left) and 88 normal samples (right) revealed upregulation of *endoG* mRNA expression in OC patient tissues compared to normal tissues. The height of each thick bar represents the median expression level. The whiskers represent the maximum and minimum values within each sample set. (E, F) Representative confocal images showing EndoG staining with an anti‐EndoG antibody (green), Mitotracker (red), and DAPI (blue) in OSE80PC and SKOV3 cells, scale bar: 20 μm, (E) and the statistical analysis of relative EndoG expression (nuclear localization of EndoG relative to that in the whole cell) (F). The images were quantified using imagej. The means ± SEM are given for three independent experiments, ***P < 0.001, unpaired *t*‐test (F). (G) Subcellular fractionation was performed for OSE80PC and SKOV3 cells. The EndoG level was measured along with marker proteins in the WCL and cytosolic and nuclear fractions; β‐tubulin, a cytosolic marker, and PARP‐1, a nuclear marker. (H) Representative immunohistochemical staining images showing high EndoG expression in carcinoma tissues (bottom) compared to non‐carcinoma tissues (top). Anti‐EndoG antibody (green). propidium iodide (PI) scale bar: 25 μm. OC tissue microarrays were obtained from US Biomax Inc (Materials and methods).

We examined the EndoG levels in OC cells. EndoG levels were upregulated in all tested OC cell lines compared to normal ovary cells and were highest in SKOV3 cells (Fig. 2A, Fig. S1A). The *endoG* transcript was also upregulated in SKOV3 cells (Fig. 2B). The EndoG promoter is regulated via methylation of CpG by DNMT1, a DNA methyltransferase [19]. DNMT1 expression was decreased in SKOV3 cells compared to that in control cells (Fig. S1B), which might explain the upregulation of *endoG* mRNA in SKOV3 cells. Moreover, the half‐life of EndoG was much longer in SKOV3 cells than that in control cells (Fig. 2C). Therefore, the upregulation of EndoG in SKOV3 cells is due to increases in both transcription and protein stability. We assessed *endoG* mRNA expression levels in OC patients by analyzing the Gene Expression Profiling Interactive Analysis (GEPIA) database and found that *endoG* was considerably increased compared to that in normal ovary tissues (Fig. 2D).

Next, we examined whether upregulated EndoG is localized in the mitochondria. Confocal images of cells immunostained with an anti‐EndoG antibody showed more intense EndoG staining in SKOV3 cells than that in control normal cells (Fig. 2E). Surprisingly, we found that EndoG was localized in the nucleus and cytosol in SKOV3 cells, while in control cells, EndoG was observed in the cytosol, where it would normally be localized without an apoptotic stimulus (Fig. 2E). The statistical analysis revealed that the relative amount of nuclear EndoG was approximately 30% higher in SKOV3 cells than that in control cells (Fig. 2F). The subcellular fractionation results confirmed that EndoG was mainly localized in the nucleus in SKOV3 cells, unlike that in control cells (normal ovary cell line; Fig. 2G). Finally, to confirm our results in OC patient tissues, immunohistochemistry was conducted with an anti‐EndoG antibody using ovary tissue microarray slides containing both normal and malignant tissues. EndoG staining was more intense in OC patient tissues than that in normal ovary tissues, and EndoG was observed throughout the whole cell, not only in the cytosol (Fig. 2H). Taken together, these results indicate that EndoG is upregulated in OC cells and patient tissues, and a considerable amount of EndoG is localized in the nucleus under normal conditions without apoptotic stress. EndoG is a cell death effector that is exclusively localized in the mitochondria and translocates to the nucleus after apoptotic stimulation, thus cleaving chromosomal DNA. Therefore, these results imply that nuclear‐localized EndoG might have a different function from that in the mitochondria. This finding may explain why EndoG depletion in SKOV3 cells resulted in decreased cell viability upon oxidative stress compared to that in control cells (Fig. 1).

### High ROS and cellular inhibitor of apoptosis protein (cIAP1) levels induce upregulation and nuclear localization of EndoG in OC cells

**Fig. 3:**
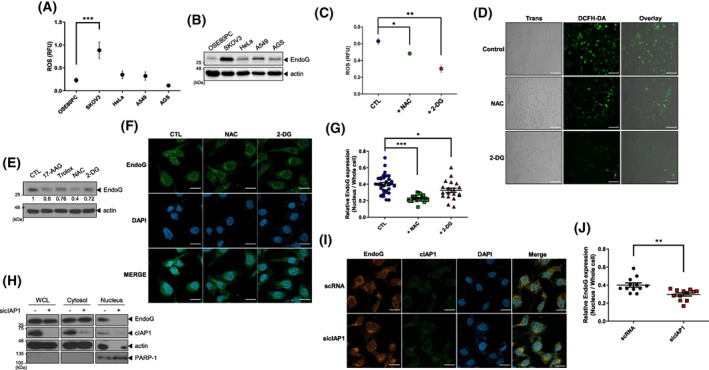
High ROS and cIAP1 levels upregulate and increase nuclear localization of EndoG in OC cells. (A, B) The OSE80PC, SKOV3, HeLa (cervical cancer), A549 (lung cancer), and AGS (gastric cancer) cell lines were incubated with 5 μm DCFH‐DA for 5 min. Fluorescence was measured using a microplate reader (Materials and methods) (A). WB analysis in a parallel experiment showed EndoG upregulation only in SKOV3 cells (B). (C, D) SKOV3 cells were treated with 10 mm NAC or 10 mm 2‐DG. After 24 h, intracellular ROS levels were measured with a microplate reader (C) and a fluorescence microscope, scale bar: 100 μm (D). (E) EndoG levels in SKOV3 cells were decreased after treatment with various antioxidants. The numbers indicate EndoG expression relative to that of Actin. (F, G) SKOV3 cells were treated with 5 mm NAC or 5 mm 2‐DG for 24 h and then immunostained with an anti‐EndoG antibody (green) and DAPI (blue). EndoG expression (F) was quantified using imagej, scale bar: 20 μm. Total EndoG expression was downregulated and the nuclear EndoG ratio was further reduced (G). (H–J) SKOV3 cells were transfected with scRNA or sicIAP1 for 48 h. The cytosolic or nuclear fraction was isolated, and each cell lysate was analyzed by WB with anti‐EndoG, anti‐cIAP1, antiactin, and anti‐PARP‐1 antibodies (H). The cells were immunostained with anti‐EndoG (red) and anti‐cIAP1 (green) antibodies and DAPI (blue), scale bar: 20 μm (I). The ratio of nuclear localization of EndoG to that in the whole cell was decreased according to analysis with imagej software (J). Values represent means ± SEM from triplicate independent experiments (***P < 0.001, **P < 0.01, *P < 0.05, unpaired *t*‐test).

Previously, we reported that EndoG expression is increased under oxidative stress [17]. Therefore, we examined whether ROS levels were elevated in SKOV3 cells, which might cause EndoG upregulation. We assessed ROS levels in normal ovary cells, SKOV3 cells, and other types of cancer cells via DCFH‐DA measurement. ROS levels were much higher in SKOV3 cells than in normal ovary cells and other cancer cells (Fig. 3A), which is consistent with several reports demonstrating high ROS levels in OC [7, 8]. Comparison of the EndoG expression in these cell lines showed correlations between EndoG and ROS levels in each cell line (Fig. 3A,B). We hypothesized that if high ROS levels caused EndoG upregulation, decreasing ROS levels in SKOV3 cells might downregulate EndoG expression. Indeed, we found that treatment with antioxidants, including N‐acetyl cysteine (NAC) or the glucose analog 2‐deoxy‐D‐glucose (2‐DG), decreased ROS levels in SKOV3 cells (Fig. 3C,D). In parallel, EndoG levels were reduced (Fig. 3E) by these antioxidants. Moreover, we found that the relative amount of nuclear EndoG in SKOV3 cells was reduced upon NAC or 2‐DG treatment (Fig. 3F,G, Fig. S2), indicating that EndoG localization and protein levels are also affected by intracellular ROS levels.

We previously reported that cIAP1, an E3 ligase, binds and ubiquitinates EndoG without affecting intracellular levels of EndoG and EndoG‐mediated cell death [22, 23]. Thus, we hypothesized that cIAP1 might be involved in the nuclear localization of EndoG through its Ring activity. Western blot (WB) results after subcellular fractionation showed that most EndoG in cIAP1‐depleted SKOV3 cells was located in the cytosol and not in the nucleus (Fig. 3H). Immunocytochemical images after treatment with an anti‐EndoG antibody and the statistical analysis revealed that the relative amount of nuclear EndoG in cIAP1‐depleted SKOV3 cells was decreased to approximately 70% of that in control cells (Fig. 3I,J). Additionally, ectopic cIAP1 expression induced by Ub R63K caused EndoG to be mainly localized in the nucleus (Fig. S3). These data indicate that cIAP1 plays a role in the nuclear localization of EndoG. Taken together, our results demonstrate that elevated ROS and cIAP1 levels are required for upregulated expression and nuclear localization of EndoG in SKOV3 cells and suggest that post‐translational modification of EndoG, such as oxidation and/or ubiquitination, might be necessary for nuclear localization.

### The oxidation mutant EndoG P199E behaves differently from wild‐type (WT) EndoG

**Fig. 4:**
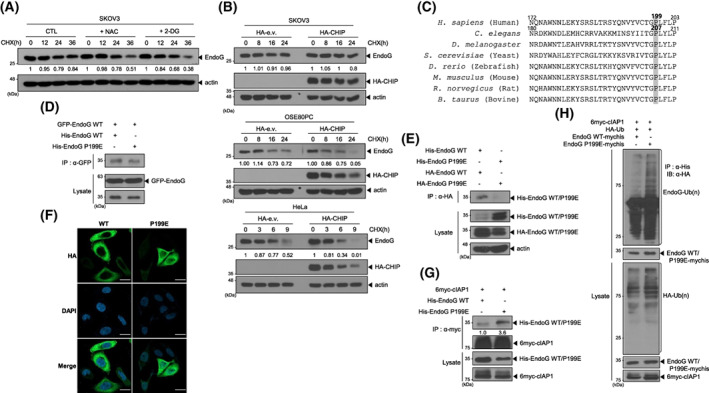
The oxidation mutant EndoG P199E behaves differently from WT EndoG. (A) Antioxidants decreased the EndoG half‐life in SKOV3 cells. The cells were co‐treated with 200 μg·mL^−1^ CHX and 10 mm NAC or 10 mm 2‐DG, and EndoG levels were compared by WB with an anti‐EndoG antibody. The numbers indicate the ratio of EndoG to Actin. (B) The EndoG half‐life was much longer in SKOV3 cells overexpressing HA‐CHIP than in OSE80PC or HeLa cells overexpressing HA‐CHIP. (C) The alignment of the EndoG sequences in various species shows their homology. (D) HEK293 cells were co‐transfected with WT GFP‐EndoG and WT His‐EndoG or His‐EndoG P199E. At 24 h after transfection, WCLs were prepared for co‐IP with an anti‐GFP antibody and then analyzed by WB using an anti‐His antibody. (E) EndoG P199E did not form a dimer in the co‐IP experiment. (F) HeLa cells were transfected with WT HA‐EndoG or HA‐EndoG P199E. The cells were immunostained with an anti‐HA antibody (green) and DAPI (blue), scale bar: 20 μm. (G) Interaction between EndoG P199E and cIAP1 was greater than that between cIAP1 and WT EndoG. (H) HeLa cells were co‐transfected with 6myc‐cIAP1, HA‐Ub, and His‐EndoG (WT or P199E). No MG132 was added to the cell culture. A ubiquitination assay was performed by co‐IP with an anti‐His antibody, and WB assays were performed with anti‐HA, anti‐His, and anti‐myc antibodies.

In addition to its enhanced expression, we examined whether EndoG stability was also altered in SKOV3 cells due to its high ROS levels. When ROS levels in SKOV3 cells were decreased by NAC or 2‐DG treatment, the half‐life of EndoG was markedly reduced (Fig. 4A). Previously, we reported that a co‐chaperone E3 ligase, C‐terminus of Hsc70‐interacting protein (CHIP), binds and ubiquitinates EndoG, targeting it for proteasomal degradation. However, under oxidative stress, CHIP and EndoG could not interact, and no EndoG ubiquitination was observed [17], implying that CHIP‐induced EndoG regulation is affected by intracellular ROS levels. Indeed, CHIP overexpression in SKOV3 cells, which have high ROS levels, did not affect the half‐life of EndoG (Fig. 4B), while CHIP overexpression resulted in a sharp decrease in the EndoG half‐life in normal ovary cells and HeLa cells (Fig. 4B). These results suggested that the conformation of EndoG might be altered by the increased ROS levels in OC. CPS‐6, an EndoG homolog in *Caenorhabditis elegans*, normally acts as a dimer; when acting as a monomer under oxidative stress, CPS6 has no nuclease activity [24]. Oxidative insult specifically oxidized P207 of CPS‐6, altering its conformation to a monomer. The primary sequence of EndoG is highly conserved from yeast to humans [11, 24]. We mutated the 199th proline of human EndoG, equivalent to Pro207 of the *C. elegans* EndoG homolog, CPS‐6, to glutamic acid (EndoG P199E, Fig. 4C) and determined whether it acts as a monomer. His‐EndoG P199E protein did not interact with WT green fluorescent protein (GFP)‐EndoG (Fig. 4D). In co‐immunoprecipitation (co‐IP) experiments, WT EndoG protein formed dimers with EndoG with various tags, but the EndoG P199E mutant protein did not form dimers (Fig. 4E). Additionally, EndoG P199E was localized in the nucleus and cytosol, while WT EndoG was expressed exclusively in the cytosol of HeLa cells, which have low intracellular ROS levels (Fig. 4F). Furthermore, cIAP1 showed greater interaction with EndoG P199E than with WT EndoG (Fig. 4G), and EndoG P199E exhibited greater ubiquitination by cIAP1 than that of WT EndoG (Fig. 4H, Fig. S4). These results indicate that human EndoG P199E behaves as a monomer and is primarily localized in the nucleus, suggesting that the high ROS levels in OC might induce an oxidized, monomeric EndoG with no nuclease activity that is readily ubiquitinated due to strong interaction with cIAP1 and is eventually localized in the nucleus under normal conditions.

### EndoG depletion reduces cell viability

**Fig. 5:**
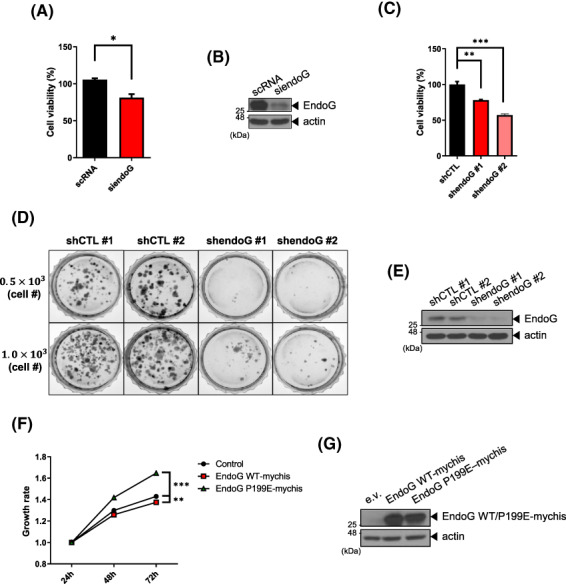
EndoG depletion reduces cell viability. (A, B) SKOV3 cells were transfected with scRNA or siendoG. At 48 h after transfection, the cell viability was analyzed by WST assay (A). WCLs were analyzed by WB with anti‐EndoG and antiactin antibodies (B). (C) EndoG‐KD cells also showed reduced viability in the WST assay (C). (D, E) EndoG‐KD cells were grown in the medium containing puromycin for 2 weeks. Colonies were stained with crystal violet, photographed (D), and subjected to a WB assay (E). (F, G) EndoG‐KD cells were transfected with EndoG WT‐mychis, HA‐EndoG P199E‐mychis, or a control empty vector. A WST assay was performed at the indicated time points after transfection (F). EndoG expression was revealed by WB (G). Values represent means ± SEM from triplicate independent experiments (***P < 0.001, **P < 0.01, *P < 0.05 unpaired *t*‐test).

Because EndoG digests chromosomal DNA in the nucleus during apoptosis, it is surprising that OC cells survive and have a considerable amount of nuclear EndoG (Figs 2, 3, 4). Thus, we determined whether EndoG plays a role in cell viability in OC cells. A WST assay showed that cells depleted of EndoG via siRNA showed approximately 80% viability compared to controls (Fig. 5A,B), and cells depleted of EndoG via shRNA showed 60–80% viability compared with controls (Figs 5C and 1D). Moreover, EndoG‐KD cells showed a marked decrease in colony numbers (Fig. 5D,E, Fig. S5). These results indicate that EndoG has a vital function in OC cells and is not involved in apoptosis. Furthermore, we overexpressed WT EndoG and EndoG P199E in EndoG‐KD cell lines and performed WST assays to determine whether increased EndoG expression enhanced cell proliferation. Cells overexpressing EndoG P199E exhibited nearly 30% enhanced cell viability compared with control cells, while the viability of cells overexpressing WT EndoG was similar to that of control cells (Fig. 5F,G), suggesting that, under high ROS conditions, EndoG monomers might produce greater cell proliferation than EndoG dimers.

### Depletion of EndoG caused cell cycle delay in the G2/M phase in OC cells

**Fig. 6:**
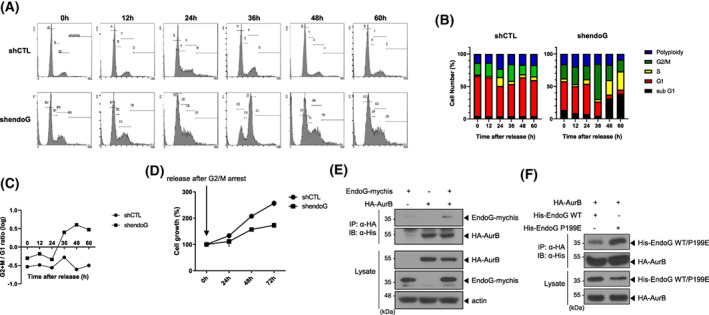
Depletion of EndoG causes a cell cycle delay in the G2/M phase in OC cells. (A–C) EndoG‐KD cells (shendoG #2) and control cells (shCTL) were arrested at the G1 phase by serum starvation for 72 h, and then the cells were released. The cell cycle was analyzed by flow cytometry (Materials and Methods). (D) Control cells (shCTL) or EndoG‐KD cells were arrested at the G2/M phase by treatment with 200 ng·mL^−1^ nocodazole for 16 h, and then the cells were released. The cell viability was measured by WST assay at the indicated time points over 72 h. (E) EndoG interacted with AurB. HEK293 cells were co‐transfected with EndoG‐mychis and HA‐AurB. After 24 h, co‐IP was performed with an anti‐HA antibody, and binding of EndoG to AurB was revealed by WB with an anti‐His antibody. (F) Comparison of WT EndoG and EndoG P199E binding to AurB. HA‐AurB showed stronger binding to His‐EndoG P199E than to WT EndoG.

Next, we aimed to understand how EndoG participates in cell proliferation. To investigate whether EndoG is involved in cell cycle progression, we synchronized control cells (shCTL) and EndoG‐KD cells (shendoG) at the G1 phase and released them after 72 h. The cell cycle profiles were monitored at the indicated time points via fluorescence‐activated cell sorting analysis. EndoG‐KD cells required almost twice the amount of time for cell doubling as that of control cells (Fig. 6A). Additionally, EndoG‐KD cells had a smaller G1 and a greater G2/M population than control cells (Fig. 6B,C). We examined whether this extended G2/M phase had a substantial effect on the progression of mitosis. Indeed, we found that EndoG‐KD cells had a greater polyploid population (Fig. S5A), and approximately fourfold more EndoG‐KD cells than control cells at the subG1 phase (Fig. S5B). In addition, we synchronized shCTL and EndoG‐KD cells at the G2/M phase using nocodazole and counted cell numbers for 72 h. The control cells doubled within nearly 48 h. However, EndoG‐KD cells showed only an approximately 50% growth increase at 72 h (Fig. 6D). We also observed similar results after G1 arrest (Fig. S5C). These results suggest that lack of EndoG expression delays cell cycle progression, characterized by an extended G2/M phase.

Aurora B (AurB) is a catalytic component of the chromosome passenger complex (CPC) and plays a key role in chromosome segregation and cytokinesis during mitosis [25]. According to proteomic analysis, AurB interacts with EndoG [26]. Therefore, we hypothesized that EndoG might participate in cell cycle regulation by interacting with AurB. Indeed, EndoG interacted with AurB (Fig. 6E, Fig. S5D), and interestingly, EndoG P199E exhibited enhanced interaction with AurB (Fig. 6F), implying that under high ROS conditions, EndoG monomers might preferentially bind to AurB, thereby regulating the mitotic process. Our results support this idea because EndoG P199E enhanced the cell proliferation rate (Fig. 5F), and EndoG‐KD cells showed an abnormal cell cycle with an extended G2/M phase and polyploidy, similar to the phenotype observed in AurB‐depleted cells (Fig. 6A–C, Fig. S5A).

### Decreasing the ROS level reduces OC cell proliferation and enhances cisplatin efficacy by reducing EndoG availability

**Fig. 7:**
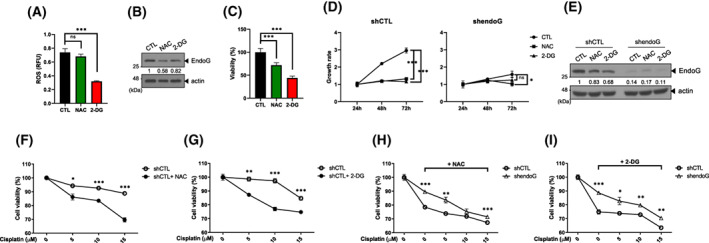
Decreased ROS levels reduce OC cell proliferation and enhance the effect of cisplatin depending on EndoG availability. (A–C) SKOV3 cells were treated with 5 mm NAC or 5 mm 2‐DG for 24 h. Then, the cells were incubated with 5 μm DCFH‐DA for 5 min. ROS levels were measured using a microplate reader (A). Changes in EndoG levels were confirmed by WB with an anti‐EndoG antibody (B). A WST assay indicated that the cell viability was decreased by NAC or 2‐DG treatment (C). (D, E) Control cells (shCTL) and EndoG‐KD cells (shendoG) were treated with 5 mm NAC or 5 mm 2‐DG, and the cell number was counted at 24–72 h (D). WB analysis showed that NAC and 2‐DG were only effective in controlling cells by reducing EndoG levels (E). (F, G) Control (shCTL) cells were treated with increasing concentrations of cisplatin in the absence or presence of NAC (F) or 2‐DG (G), and cell viability was measured by WST assay. (H, I) The response to co‐treatment with cisplatin and NAC (H) or 2‐DG (I) was compared between the control cells (shCTL) and EndoG‐KD cells (shendoG) using a WST assay. Values represent means ± SEM from triplicate independent experiments (***P < 0.001, **P < 0.01, *P < 0.05, unpaired *t*‐test).

Our findings showed that increased ROS levels upregulated EndoG and altered its conformation, enhancing OC cell proliferation. Thus, we examined whether decreasing intracellular ROS levels would decrease cell viability due to a change in EndoG. If so, modulation of ROS levels in OC might be a promising strategy to suppress OC cell growth. Furthermore, NAC or 2‐DG treatment reduced intracellular ROS levels (Fig. 7A) and endogenous EndoG levels (Fig. 7B), consistent with the results shown in Fig. 3. In parallel, cell viability was decreased by NAC or 2‐DG treatment (Fig. 7C). If our hypothesis was true, EndoG‐KD cells would not exhibit reduced growth under low ROS levels. NAC or 2‐DG treatment inhibited growth in control cells (shCTL). However, untreated EndoG‐KD cells exhibited little cell growth, and antioxidant treatment did not further suppress growth (Fig. 7D). The EndoG level reduction was confirmed by WB analysis in a parallel experiment (Fig. 7E). These results suggest that OC cell growth depends on the availability of EndoG, which is quantitatively and qualitatively modulated by the intracellular ROS concentration.

We hypothesized that decreasing the ROS level could improve cisplatin efficacy, especially in EndoG‐upregulated cancers such as OC. Indeed, the cell viability of control cells (shCTL) was markedly decreased by combined treatment with NAC and increasing concentrations of cisplatin compared to treatment with cisplatin alone (Fig. 7F). Combined treatment with 2‐DG and cisplatin also showed a similar result (Fig. 7G). However, a clear discrepancy in EndoG‐mediated cell viability suppression was observed between EndoG‐KD cells (shendoG) and control cells (shCTL) treated with antioxidants and cisplatin (Fig. 7H,I). Control cells with endogenous EndoG were much more sensitive to NAC (Fig. 7H) and 2‐DG (Fig. 7I), resulting in improved efficacy of low‐dose cisplatin. However, EndoG‐KD cells were less sensitive to antioxidants, as shown in Fig. 7D, and no obvious combined effect was observed. Taken together, our results demonstrate that antioxidants can at least partially enhance cisplatin efficacy by targeting EndoG in anti‐OC therapy.

## Discussion

### EndoG is not only a DNA‐cleaving nuclease and a cell death effector but is also a multifunctional protein modulated by intracellular ROS dynamics

During the two decades since EndoG was shown to be a cell death effector in *C. elegans* and mice, most studies have focused on inducing EndoG activity to kill cancer cells [20, 21, 27] or blocking its activity to induce cytoprotection [28, 29]. In this classical view, EndoG is primarily localized in mitochondria during translation and translocates to the nucleus upon apoptotic insult. However, a growing number of studies have shown nonapoptotic roles of EndoG in either mitochondria or the nucleus. EndoG is involved in the maintenance of mitochondria [30, 31], conservative recombination [32], and DNA rearrangement upon replication stress in the nucleus [33]. These reports extend the classical view of EndoG, especially regarding its nonapoptotic function in the nucleus. EndoG was originally purified and characterized in mammalian nuclei [34]. Therefore, it is not clear why EndoG does not cleave chromosomal DNA but rather plays a different role in the nucleus. High ROS levels have been shown to affect the conformation of purified CPS‐6 (a homolog of human EndoG) in *C. elegans*. Under oxidative stress, CPS‐6 undergoes structural alteration by oxidation, and EndoG monomers with diminished nuclease activity, rather than EndoG dimers, are present, inhibiting cell death [24]. Thousands of proteins can be reversibly oxidized under high ROS conditions, especially by H_2_O_2_ [35]. Therefore, we hypothesized that intracellular oxidative stress represented by high ROS levels might modulate the conformation of human EndoG. Most cancer cells have high ROS levels due to their high energy requirements [7]. Indeed, high intracellular ROS levels are maintained in OC cell lines and patient tissues [8] (Fig. 3), and nuclear EndoG plays a vital role in OC proliferation (Figs 2, 3, 4, 5, 6, 7). We assumed that in OC cells, which have high ROS levels, EndoG behaves as a monomer and is primarily oxidized and ubiquitinated by cIAP1, showing no nuclease activity. We have previously shown that increased EndoG levels during oxidative stress induce caspase‐independent cell death in HeLa cells and primary rat cortical neurons [17]. Thus, it is unclear why EndoG shows the opposite response under high ROS conditions. OC cells may adapt to high ROS conditions, and therefore post‐translational modifications of EndoG, such as oxidation and ubiquitination, would primarily affect EndoG expression, nuclear localization, and, subsequently, cell proliferation. Upregulation of EndoG has been shown in gastric and colorectal cancer patient tissues via immunohistochemistry [36]. The proposed mechanism in this study is likely to at least partly contribute to tumorigenesis in gastric and colorectal cancer through the upregulation of EndoG.

It is not clear how EndoG is regulated in normal cells if cancer cells are an extreme example. Cells dynamically maintain intracellular ROS within a certain range. Therefore, EndoG might be modified through ROS dynamics, and thus structurally altered types of EndoG could co‐exist, with some localized in the nucleus and others localized in the mitochondria or cytosol. These EndoG subtypes could play distinct, either nonapoptotic or apoptotic, roles according to changes in ROS dynamics.

### Nuclear EndoG is involved in OC cell cycle regulation

The role of upregulated nuclear EndoG in OC is unclear. We show that EndoG plays a vital role in cell proliferation because EndoG depletion reduced cell growth and overexpression of EndoG P199E‐stimulated cell growth in EndoG‐KD cells (Figs 5 and 6). Similarly, reduced EndoG expression has been shown to decrease cell viability in human embryonic kidney 293T (HEK293T) cells [37]. Additionally, a homolog of EndoG in yeast, Nuc1, exerts dual functionality, playing either a lethal or vital role, depending on mitochondrial respiration [38]. EndoG‐depleted cells had a delayed cell cycle, especially at the G2/M phase, and an increased polyploid population (Fig. 6, and Fig. S5A,B). These results imply that EndoG is involved in cell cycle progression, especially in the late step of mitosis, because polyploidy indicates failure of cytokinesis. Chromosome segregation and cytokinesis are late steps during the completion of mitosis. These events are mainly controlled by the CPC, which is composed of AurB, INCENP, Survivin, and Borealin [25]. AurB plays a key role in chromosome segregation by correcting kinetochore attachment errors [25]. AurB was originally identified in yeast when screening for increase‐in‐ploidy mutants [39], and dysregulation of its activity caused aneuploidy in mammals [40]. We observed that AurB interacts with EndoG and has greater interaction with the EndoG P199E mutant (Fig. 6), suggesting that EndoG is involved in the regulation of mitosis and supports the function of AurB. Moreover, cIAP1, a member of the Inhibitor of Apoptotic Proteins family, shares baculoviral IAP repeat (BIR) domains [22] and binds and ubiquitinates EndoG P199E and WT EndoG, and cIAP1 depletion decreases nuclear EndoG localization (Figs 3H–J and 4H,G). Considering that EndoG binds to the BIR domains of cIAP1 [23], EndoG might interact with Survivin, a component of the CPC and a member of the IAP family with a single BIR domain. Therefore, we postulate that nuclear EndoG might augment the formation of CPC through its binding to AurB and potentially to Survivin.

### Targeting EndoG alone or in combination with platinum‐type agents could be effective for OC therapy

We suggest that the key to cancer treatment is to discover altered genes and understand their mechanism of action in tumorigenesis. We found that in OC, EndoG is upregulated, and EndoG localized in the nucleus has a nonapoptotic function (Figs 1, 2, 3, 4). In OC, elevated ROS upregulates transcription and protein maintenance of EndoG (Figs 3 and 4). Therefore, we propose that the EndoG molecule is a reasonable candidate for the development of specific anti‐OC therapy. An inhibitor that directly bound to EndoG was demonstrated by chemical library screening [28]. Thiobarbiturate analogs inhibited the nuclease activity of EndoG in the dimer conformation, showing a cytoprotective effect against various apoptotic stimuli.

However, it is unlikely that this type of inhibitor would effectively inhibit the altered EndoG under elevated ROS conditions. Another EndoG‐targeting method to consider is antioxidant treatment. SOD1‐overexpressing transgenic rats exhibited reduced oxidative stress, and significantly less EndoG translocated to the nucleus [41]. We demonstrated that decreasing intracellular ROS levels downregulated EndoG, especially in the nucleus of OC cells (Fig. 3). Various types of antioxidants have been used in pre‐clinical and clinical research, including NADPH oxidase inhibitors, SOD mimetics, and NAC [reviewed in ref. 9]. Among them, NAC has been used in combination with standard chemotherapy. NAC treatment potentiated doxorubicin‐induced ATM and p53 phosphorylation, thereby enhancing their inhibition of cell proliferation in OC [42]. We demonstrated that NAC treatment decreased intracellular ROS, downregulating EndoG and decreasing cell viability (Figs 3 and 7). Furthermore, NAC sensitized OC cells to cisplatin in an EndoG‐dependent manner (Fig. 7). The glucose analog 2‐DG competes with glucose, interfering with aerobic glycolysis in cancer, and thus is widely used because of its antitumor potential. Additionally, 2‐DG has been shown to increase intracellular ROS, thereby facilitating the apoptosis of certain types of cancer cells [43]. However, interestingly, we observed that 2‐DG treatment significantly decreased ROS levels in OC cells, which were sensitized to cisplatin due to the reduction of EndoG (Fig. 7).

In our study, we demonstrated that elevated ROS in OC cell lines induces EndoG expression and nuclear localization, playing a vital role in OC tumorigenesis. We propose that targeting EndoG, either by itself or in combination with platinum‐type agents, might be a promising strategy to enhance cancer cell death in the treatment of OC patients. This strategy can also be considered for the treatment of cancers with high EndoG expression such as gastric or colorectal cancers.

## Materials and methods

### Cell culture, transfection, and RNA interference

The ovarian cancer cell lines used in this study were acquired from Korean Cell Line 13 Bank. SKOV3, A2780, and OVCAR3 cell lines were cultured in RPMI (Welgene, Seoul, Korea) supplemented with 5% or 10% fetal bovine serum (FBS) at 37 °C in a humidified 5% CO₂ atmosphere. The HeLa cervical cancer cell line and HEK293 cells were cultured in DMEM (Welgene) supplemented with 10% FBS at 37 °C in a humidified 5% CO₂ atmosphere. The stable OC cell lines derived from SKOV3 cells generated in this study were maintained in the presence of puromycin. Transfection was performed using PEI (Sigma‐Aldrich Inc, St. Louis, MO, USA) or Lipofectamine 2000 (Invitrogen, Carlsbad, CA, USA) according to the manufacturer's instructions. *cIAP1* or *endoG* siRNA was transfected into SKOV3 cells using Lipofectamine 2000 (Invitrogen) according to the manufacturer's instructions. After 48 h, whole cell lysates (WCLs) were analyzed by WB. The siRNA sequences were as follows (sense strand, 5′ to 3′): *cIAP1*, UCGCAAUGAUGAUGUCAAA; *endoG*, GGAAAUCCUACGUAAAGUA.

### Antibodies and co‐IP

Anti‐EndoG (Proteintech, Rosemont, IL, USA), antiactin (Bethyl, Montgomery, TX, USA), anti‐HA (Covance, Emeryville, CA, USA), anti‐PARP‐1 (Santa Cruz Biotechnology, Inc. Dallas, TX, USA), anti‐His, anti‐GFP, anti‐β‐tubulin, anti‐myc 9E10 (Millipore, Billerica, MA, USA), and anti‐cIAP1 (R&D Systems, Minneapolis, MN, USA) were used for WB or co‐ IP assays.

Co‐IP was performed as follows unless otherwise noted. Cells were lysed in lysis buffer (50 mm Tris pH 8.0, 150 mm NaCl, 1 mm EDTA, 10% glycerol, 1% Triton X‐100, protease inhibitor cocktail). WCLs were mixed with the indicated antibody for 2 h at 4 °C. Protein‐A sepharose beads (Sigma‐Aldrich) were incubated with the immunocomplex for 2 h at 4 °C and then washed three times with IP wash buffer (20 mm Tris pH 8.0, 150 mm NaCl, 1 mm EDTA, 1% Triton X‐100). Samples were subjected to sodium dodecyl‐sulfate polyacrylamide gel electrophoresis and analyzed by WB.

### Database analysis

The mRNA expression levels of *endoG* in 426 tumors and 88 normal ovarian tissue samples were analyzed using the GEPIA database (http://gepia2.cancer‐pku.cn). The GEPIA database is an interactive web server used to estimate mRNA expression data based on 9736 tumors and 8587 normal samples in The Cancer Genome Atlas and Genotype‐Tissue Expression dataset projects. All boxplot analyses are presented with log_2_ (TPM + 1) values for the log scale.

### Immunofluorescence

Cells grown on 12‐mm diameter coverslips were fixed in 4% formaldehyde in phosphate‐buffered saline (PBS) for 20 min and permeabilized using 0.5% Triton X‐100 in PBS for 2 min. Cells were blocked in 5% normal goat serum in PBS for 1 h and then incubated overnight with the indicated primary antibody. After washing with PBS, cells were incubated with anti‐rabbit Alexa Fluor 546‐ or anti‐mouse Alexa Fluor 488‐conjugated secondary antibody (Invitrogen). After washing with PBS, the cells were counterstained with diamidino‐2‐phenylindole (DAPI) for 15 min and mounted on glass slides (Vector Laboratories, Inc., Burlingame, CA, USA). MitoTracker Red CMXRos (200 nm; Invitrogen) was used to stain mitochondria. Images were visualized using an LSM 800 Meta confocal microscope (Carl Zeiss, Inc., Oberkochen, Germany) and further analyzed by imagej software for quantification.

Ovarian tissue microarrays were obtained from a commercial supplier (US Biomax, Rockville, MD, USA; TMA catalog number T113a). The array contained biospecimens from 12 OC patients and one sample of normal ovary tissue, with duplicate cores for each OC patient and normal tissue. Immunohistochemistry was performed with an anti‐EndoG antibody according to the manufacturer's instructions.

### EndoG‐KD stable cell lines and colony formation assay

Production of lentiviral particles was conducted according to the manufacturer's instructions (Sigma‐Aldrich); the packaging lentiviral vector pLKO.1 contained control shRNA (shCTL) and endoG‐specific shRNA (shEndoG, #1: sigma TRCN0000039644 and #2: sigma TRCN0000039645, respectively). To establish stable cell lines, SKOV3 cells were seeded in 60‐mm culture dishes. Upon reaching 70% confluence, lentiviral particles at a multiplicity of infection of 15 in culture medium were added to the cells in the presence of 8 μg·mL^−1^ hexadimethrine bromide (Sigma‐Aldrich), which increases the efficiency of viral infection. At 24 h after infection, puromycin (2 μg·mL^−1^) was added for selection for 72 h. EndoG‐KD cell lines were seeded in six‐well plates after maintenance at 37 °C, 5% CO₂ for 2 weeks. The colonies formed by each cell were fixed with 4% formaldehyde for 30 min and stained with 0.2% crystal violet for 40 min. The assay was performed in triplicate. All the experimental protocols, usage of human cell lines, and chemicals have been approved by the Institutional Biosafety Committee (IBC) at Kyung Hee University.

### Protein half‐life measurement

OSE80PC, SKOV3, and HeLa cells were treated with 200 μg·mL^−1^ cycloheximide (CHX) and then harvested at the indicated time points. Cells were lysed with RIPA buffer (50 mm Tris pH 7.4, 150 mm NaCl, 0.1% sodium dodecyl‐sulfate, 1% Triton X‐100, 0.5% Na‐deoxycholate, 1 mm EDTA), and each sample was subjected to WB analysis.

### Quantitative reverse‐transcription polymerase chain reaction (qRT‐PCR) analysis

Total RNA was isolated using an easy‐spin RNA extraction kit (iNtRON, Seongnam‐Si, Korea) according to the manufacturer's instructions. cDNA synthesis was performed using an M‐MLV cDNA synthesis kit (Enzynomics, Daejeon, Korea). qRT‐PCR was performed using a 2x SensiFAST SYBR No‐ROX Mix (BIOLINE, Memphis, TN, USA) according to the manufacturer's instructions. The primer sets were as follows: *endoG* (sense: 5′‐CGACACGTTCTACCTGAGCA‐3′, antisense: 5′‐AGGATTTCCCATCAGCCTCT‐3′), *Dnmt1* (sense: 5′‐TACCTGGACGACCCTGACCTC‐3′, antisense: 5′‐CGTTGGCATCAAAGATGGACA‐3′), and glyceraldehyde 3‐phosphate dehydrogenase (*GAPDH*, sense: 5′‐GAGTCAACGGATTTGGTCGT‐3′, antisense: 5′‐TTGATTTTGGAGGGATCTCG‐3′).

### Cell fractionation

The cells were lysed in Buffer A (10 mm HEPES, 10 mm KCl, 0.1 mm EDTA, 0.1 mm EGTA, 1 mm DTT, 0.5 mm PMSF, protease inhibitor cocktail) and incubated for 15 min on ice. Buffer B (10% NP‐40) was added, and the cells were centrifuged at 20 160 *g* for 10 min at 4 °C. The supernatant (cytosolic fraction) was then transferred to a clean tube. The pellet was washed twice with ice‐cold Buffer A and resuspended in Buffer C (20 mm HEPES pH 7.9, 0.4 m NaCl, 1 mm EGTA, 1 mm DTT, 1 mm PMSF, protease inhibitor cocktail). The pellet was vortexed on the highest setting for 30 min at 4 °C. The supernatant (nuclear fraction) was transferred to a clean tube, and each sample was subjected to WB analysis.

### Cell viability assay

The cells were seeded in 96‐well plates, and cell viability was assessed using the EZ‐cytox kit (Dogen Bio, Seoul, Korea) according to the manufacturer's instructions. The absorbance was measured at 450 nm using Multi‐Mode microplate readers (BioTek, Winooski, VT, USA). The assay was performed in triplicate.

### ROS measurement

The cells were seeded in 96‐well black plates. DCFH‐DA (2′, 7′‐dichlorofluorescein diacetate, 5 μm) was pre‐incubated in DPBS (DCFH‐DA working solution) at 37 °C for 30 min. The cells were washed twice with DPBS and then incubated in 100 μL DCFH‐DA working solution at 37 °C for 5 min. Fluorescence was measured by a fluorescence microplate reader (Molecular Devices, San Jose, CA, USA). The assay was performed in triplicate.

### Flow cytometry

The cell lines were trypsinized and then centrifuged at 108 *g*. The cells were washed twice with DPBS, fixed with 75% ethanol for 1 h at 4 °C, and then washed twice with DPBS. Harvested cells were resuspended in propidium iodide (PI) staining solution (3.8 mm sodium citrate, 50 μg·mL^−1^ PI in PBS). Fluorescence was analyzed using an FC500 flow cytometer (Beckman Coulter Inc., Miami, FL, USA).

## Conflict of interest

The authors declare no conflict of interest.

## Author contributions

YNC and TWS designed and performed most of the experiments. YTL performed the experiments and the data analysis and helped to write the manuscript. DHJ assisted in some experiments and data analysis. SJY designed the experiments, supervised the project, and wrote the manuscript. All authors read and approved the manuscript.

## Supporting information


**Fig. S1.** The levels of EndoG in various ovarian cancer cell lines. (A) The steady‐state levels of EndoG were analyzed by WB with an anti‐EndoG antibody in OSE80PC cells and the ovarian cancer cell lines JH514, OVCAR3, and SKOV3. The numbers indicate expression of EndoG relative to actin. (B) mRNA expression of *dnmt1* via RT‐PCR in normal ovary cells (OSE80PC) or ovarian cancer cells (SKOV3). GAPDH was used as an internal control for RT‐PCR.
**Fig. S2.** The relative nuclear EndoG level in SKOV3 cells treated with10 mM NAC or 10 mM 2‐DG (A) or with cotreatment of 5 mM NAC and 5 mM 2‐DG (B) for 24 hours. Immunostaining was performed with an anti‐EndoG antibody and DAPI, and the images and statistical analysis were performed using Image J (means ± SEM are given for three independent experiments, ***p<0.001, **p<0.01, unpaired t‐test).
**Fig. S3.** Overexpression of cIAP1 with Ub R63K led to EndoG being mainly localized in the nucleus. SKOV3 cells were transfected with 6myc‐cIAP1 and HA‐Ub R63K for 24 hours and immunostained with the indicated antibodies, scale bar: 20 μm.
**Fig. S4.** EndoG P199E exhibited greater ubiquitination by cIAP1. (A) Ubiquitination assay (Materials and Methods). (B) Statistical analysis after quantification of (A) using Image J (means ± SEM are given for three independent experiments, ***p<0.001, unpaired t‐test).
**Fig. S5.** EndoG‐KD cells (shendoG) showed more cells with tetraploidy. (A) and subG1‐staged cells (B) than control cells (shCTL) on analysis of the cell cycle profile using flow cytometry after serum starvation for72 hours. (C) The cells were arrested at the G1 phase by serum starvation, and then the cells were released. The cell viability was measured by WST assay over 72 hours, means ± SEM are given for three independent experiments. (D) HEK293 cells were co‐transfected with EndoG‐mychis and HA‐AurB. After 24 hours, co‐IP assays were performed with an anti‐myc antibody, and HA‐AurB binding to EndoG‐mychis was revealed using an anti‐HA antibody on WB.

## Data Availability

The data that support the findings of this study are available from the corresponding author [yoosoonji@khu.ac.kr] upon reasonable request.
