# Supplementary material for: Nuclear endonuclease G controls cell proliferation in ovarian cancer
Source: FEBS Open Bio. 2023 Feb 13;13(4):655–69. doi: 10.1002/2211-5463.13572 (PMC10068316; doi:10.1002/2211-5463.13572)
Supplement: Supplementary file 1 — Fig. S1. The levels of EndoG in various ovarian cancer cell lines. (A) The steady‐state levels of EndoG were analyzed by WB with an anti‐EndoG antibody in OSE80PC cells and the ovarian cancer cell lines JH514, OVCAR3, and SKOV3. The numbers indicate expression of EndoG relative to actin. (B) mRNA expression of dnmt1 via RT‐PCR in normal ovary cells (OSE80PC) or ovarian cancer cells (SKOV3). GAPDH was used as an internal control for RT‐PCR. Fig. S2. The relative nuclear EndoG level in SKOV3 cells treated with10 mM NAC or 10 mM 2‐DG (A) or with cotreatment of 5 mM NAC and 5 mM 2‐DG (B) for 24 hours. Immunostaining was performed with an anti‐EndoG antibody and DAPI, and the images and statistical analysis were performed using Image J (means ± SEM are given for three independent experiments, ***p<0.001, **p<0.01, unpaired t‐test). Fig. S3. Overexpression of cIAP1 with Ub R63K led to EndoG being mainly localized in the nucleus. SKOV3 cells were transfected with 6myc‐cIAP1 and HA‐Ub R63K for 24 hours and immunostained with the indicated antibodies, scale bar: 20 μm. Fig. S4. EndoG P199E exhibited greater ubiquitination by cIAP1. (A) Ubiquitination assay (Materials and Methods). (B) Statistical analysis after quantification of (A) using Image J (means ± SEM are given for three independent experiments, ***p<0.001, unpaired t‐test). Fig. S5. EndoG‐KD cells (shendoG) showed more cells with tetraploidy. (A) and subG1‐staged cells (B) than control cells (shCTL) on analysis of the cell cycle profile using flow cytometry after serum starvation for72 hours. (C) The cells were arrested at the G1 phase by serum starvation, and then the cells were released. The cell viability was measured by WST assay over 72 hours, means ± SEM are given for three independent experiments. (D) HEK293 cells were co‐transfected with EndoG‐mychis and HA‐AurB. After 24 hours, co‐IP assays were performed with an anti‐myc antibody, and HA‐AurB binding to EndoG‐mychis was revealed using an anti‐HA antibody o [file FEB4-13-655-s001.pptx]

## Slide 1
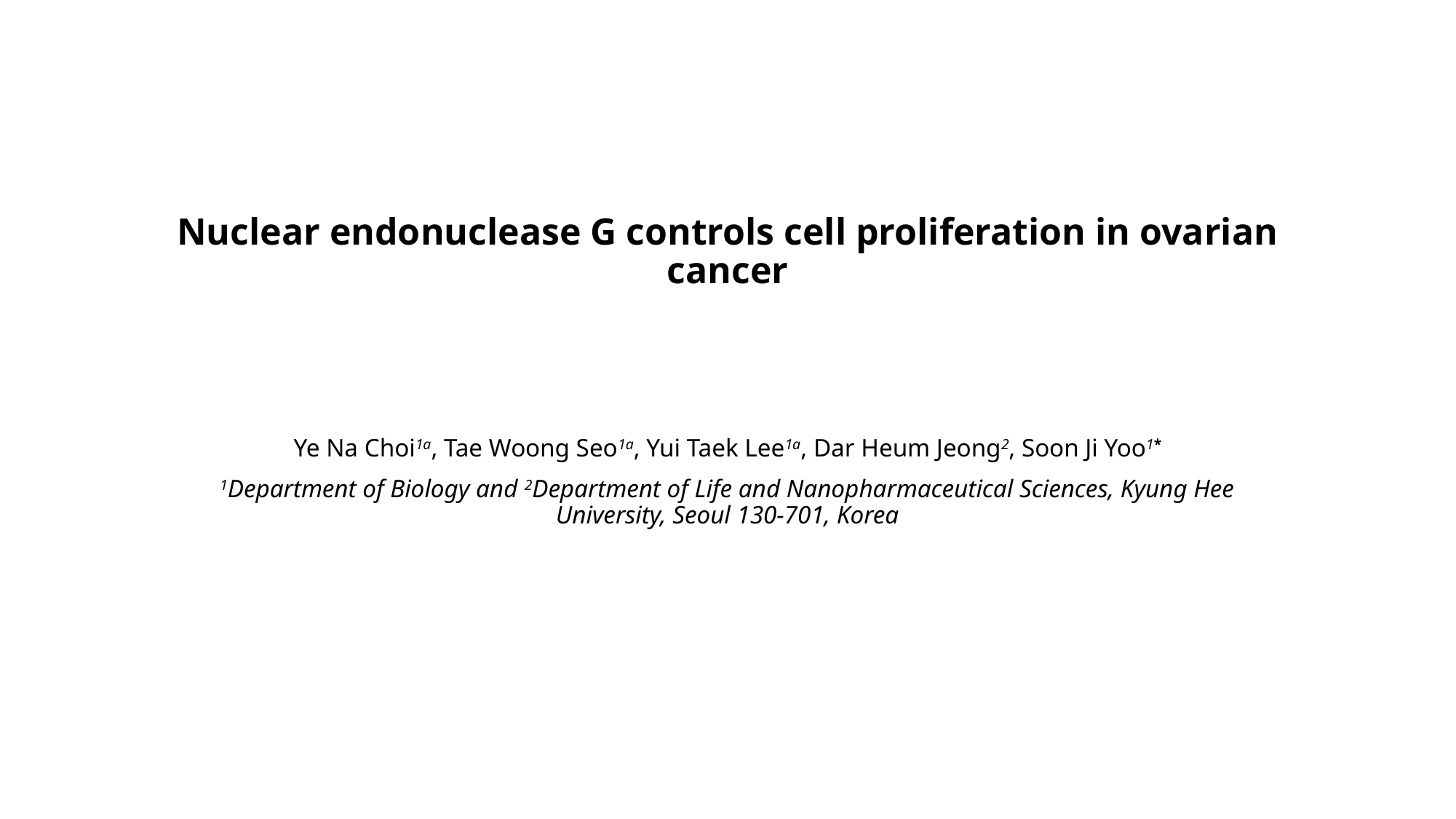

# Nuclear endonuclease G controls cell proliferation in ovarian cancer
Ye Na Choi1a, Tae Woong Seo1a, Yui Taek Lee1a, Dar Heum Jeong2, Soon Ji Yoo1*
1Department of Biology and 2Department of Life and Nanopharmaceutical Sciences, Kyung Hee University, Seoul 130-701, Korea

## Slide 2
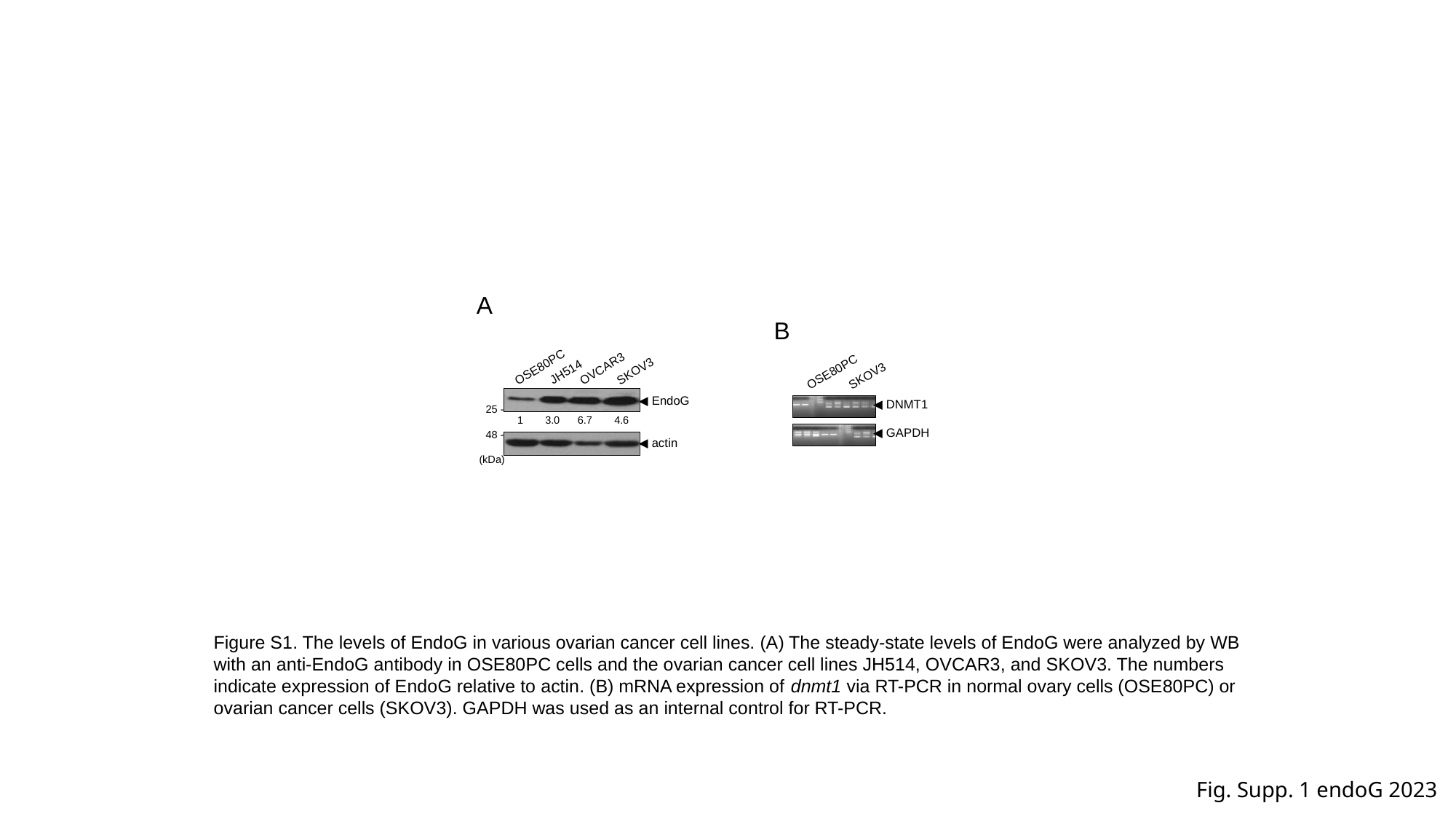

A
OSE80PC
JH514
OVCAR3
SKOV3
◀ EndoG
1
3.0
6.7
4.6
◀ actin
25 -
48 -
(kDa)
B
OSE80PC
SKOV3
◀ DNMT1
◀ GAPDH
Figure S1. The levels of EndoG in various ovarian cancer cell lines. (A) The steady-state levels of EndoG were analyzed by WB with an anti-EndoG antibody in OSE80PC cells and the ovarian cancer cell lines JH514, OVCAR3, and SKOV3. The numbers indicate expression of EndoG relative to actin. (B) mRNA expression of dnmt1 via RT-PCR in normal ovary cells (OSE80PC) or ovarian cancer cells (SKOV3). GAPDH was used as an internal control for RT-PCR.
Fig. Supp. 1 endoG 2023

## Slide 3
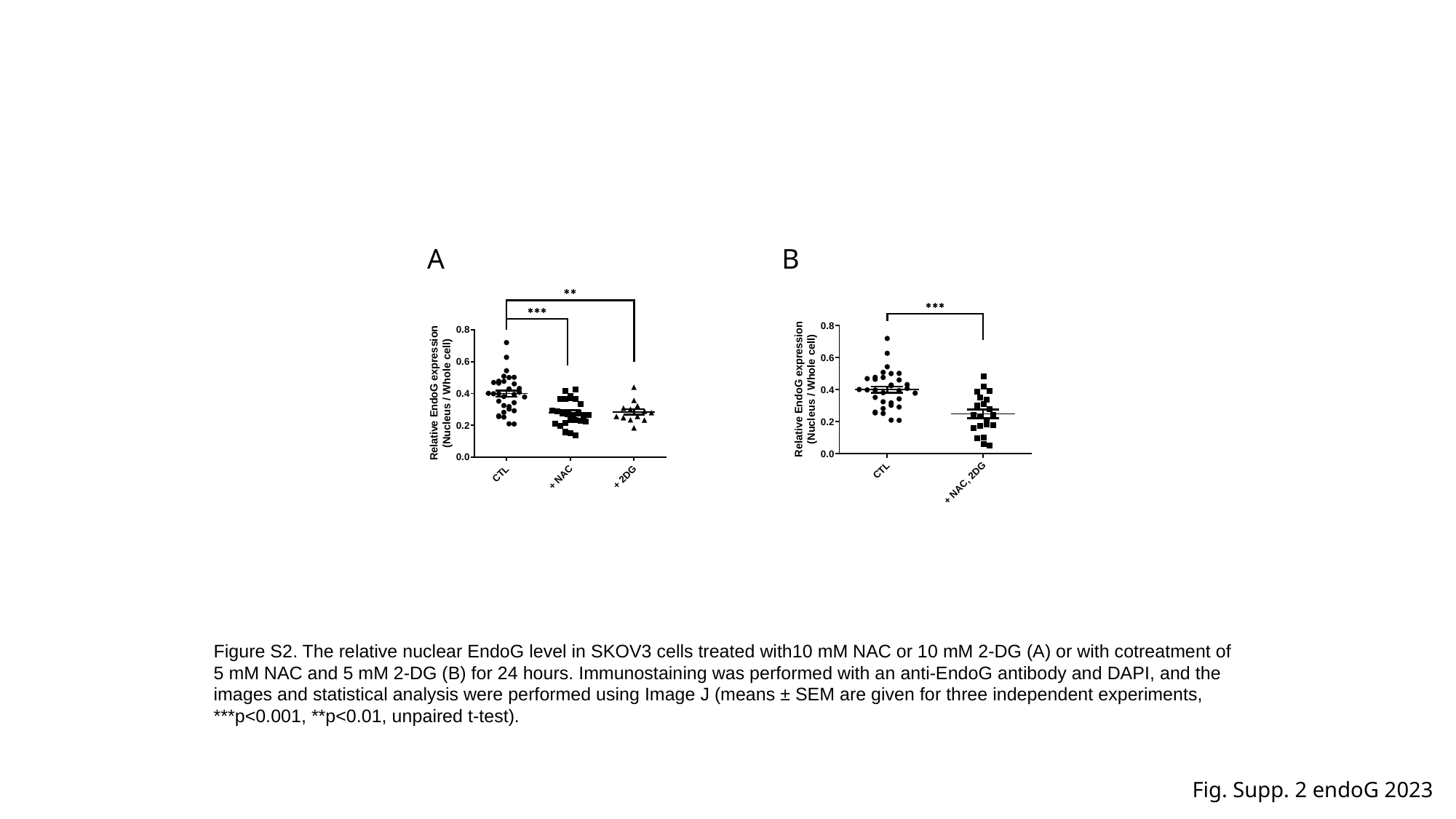

A
B
Figure S2. The relative nuclear EndoG level in SKOV3 cells treated with10 mM NAC or 10 mM 2-DG (A) or with cotreatment of 5 mM NAC and 5 mM 2-DG (B) for 24 hours. Immunostaining was performed with an anti-EndoG antibody and DAPI, and the images and statistical analysis were performed using Image J (means ± SEM are given for three independent experiments, ***p<0.001, **p<0.01, unpaired t-test).
Fig. Supp. 2 endoG 2023

## Slide 4
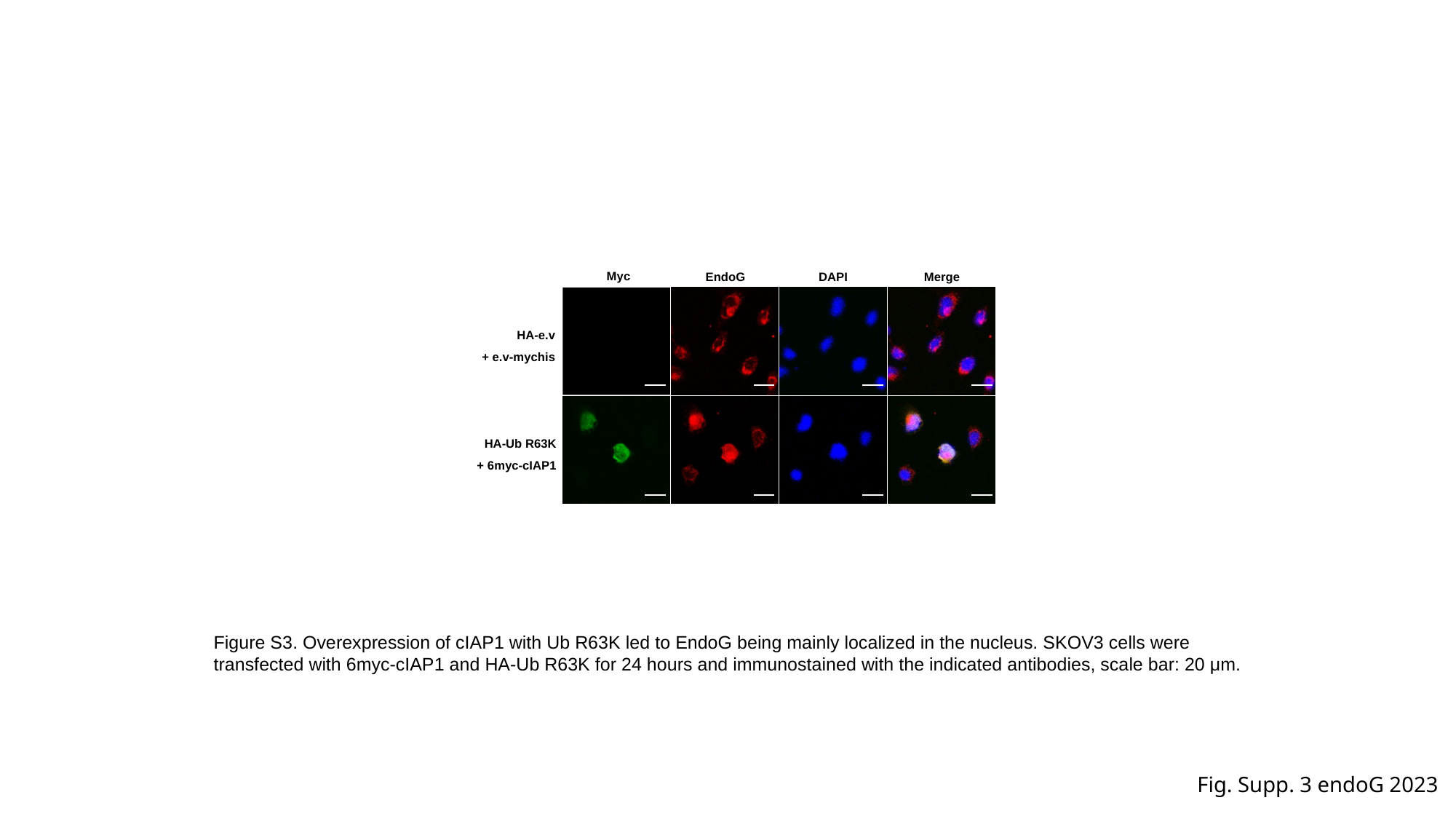

Myc
EndoG
DAPI
Merge
HA-e.v
+ e.v-mychis
HA-Ub R63K
 + 6myc-cIAP1
Figure S3. Overexpression of cIAP1 with Ub R63K led to EndoG being mainly localized in the nucleus. SKOV3 cells were transfected with 6myc-cIAP1 and HA-Ub R63K for 24 hours and immunostained with the indicated antibodies, scale bar: 20 μm.
Fig. Supp. 3 endoG 2023

## Slide 5
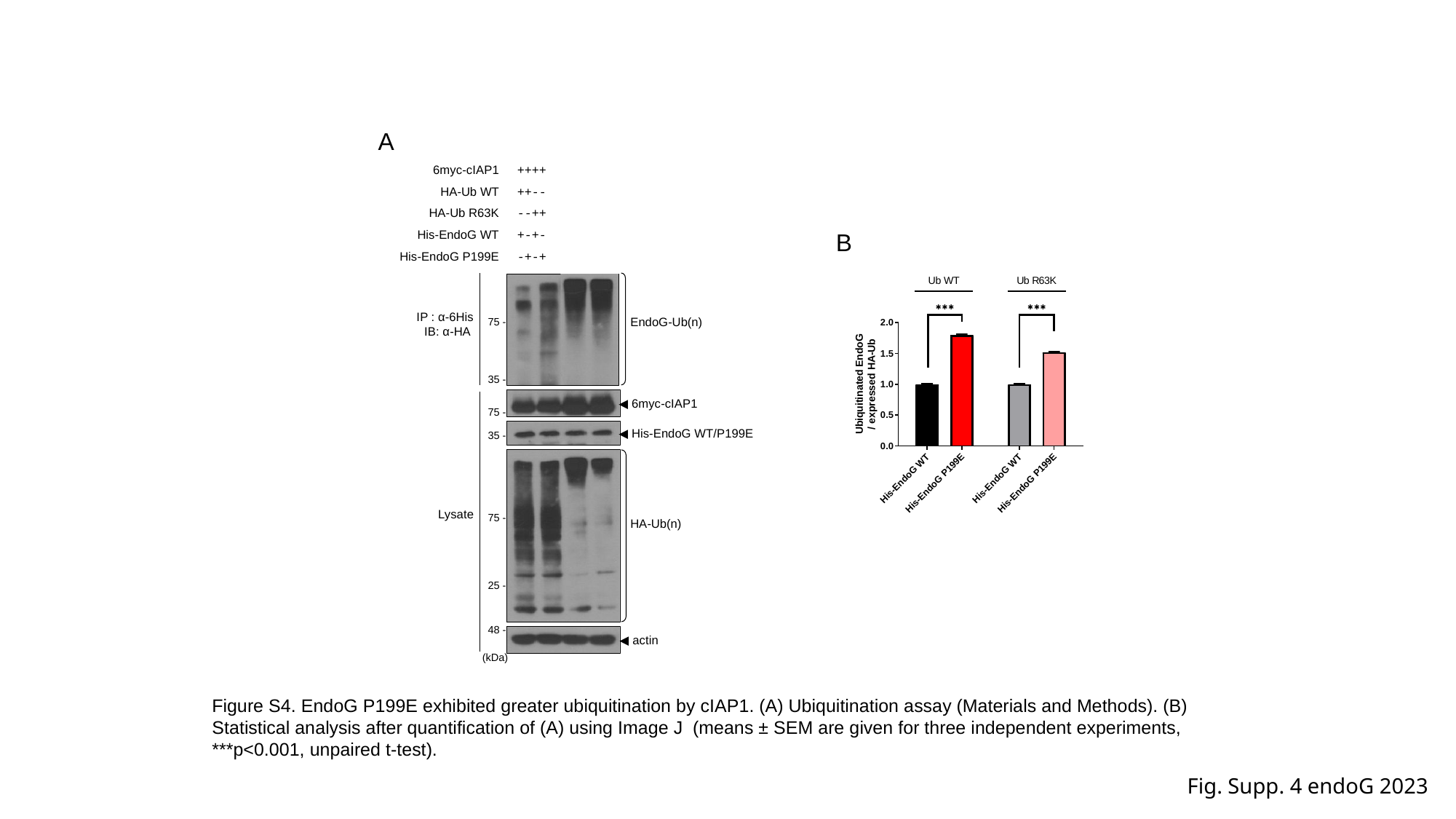

A
6myc-cIAP1
HA-Ub WT
HA-Ub R63K
His-EndoG WT
His-EndoG P199E
++++
++--
--++
+-+-
-+-+
IP : α-6His
IB: α-HA
EndoG-Ub(n)
75 -
35 -
◀ 6myc-cIAP1
75 -
◀ His-EndoG WT/P199E
35 -
Lysate
75 -
HA-Ub(n)
25 -
48 -
◀ actin
(kDa)
B
Figure S4. EndoG P199E exhibited greater ubiquitination by cIAP1. (A) Ubiquitination assay (Materials and Methods). (B) Statistical analysis after quantification of (A) using Image J (means ± SEM are given for three independent experiments, ***p<0.001, unpaired t-test).
Fig. Supp. 4 endoG 2023

## Slide 6
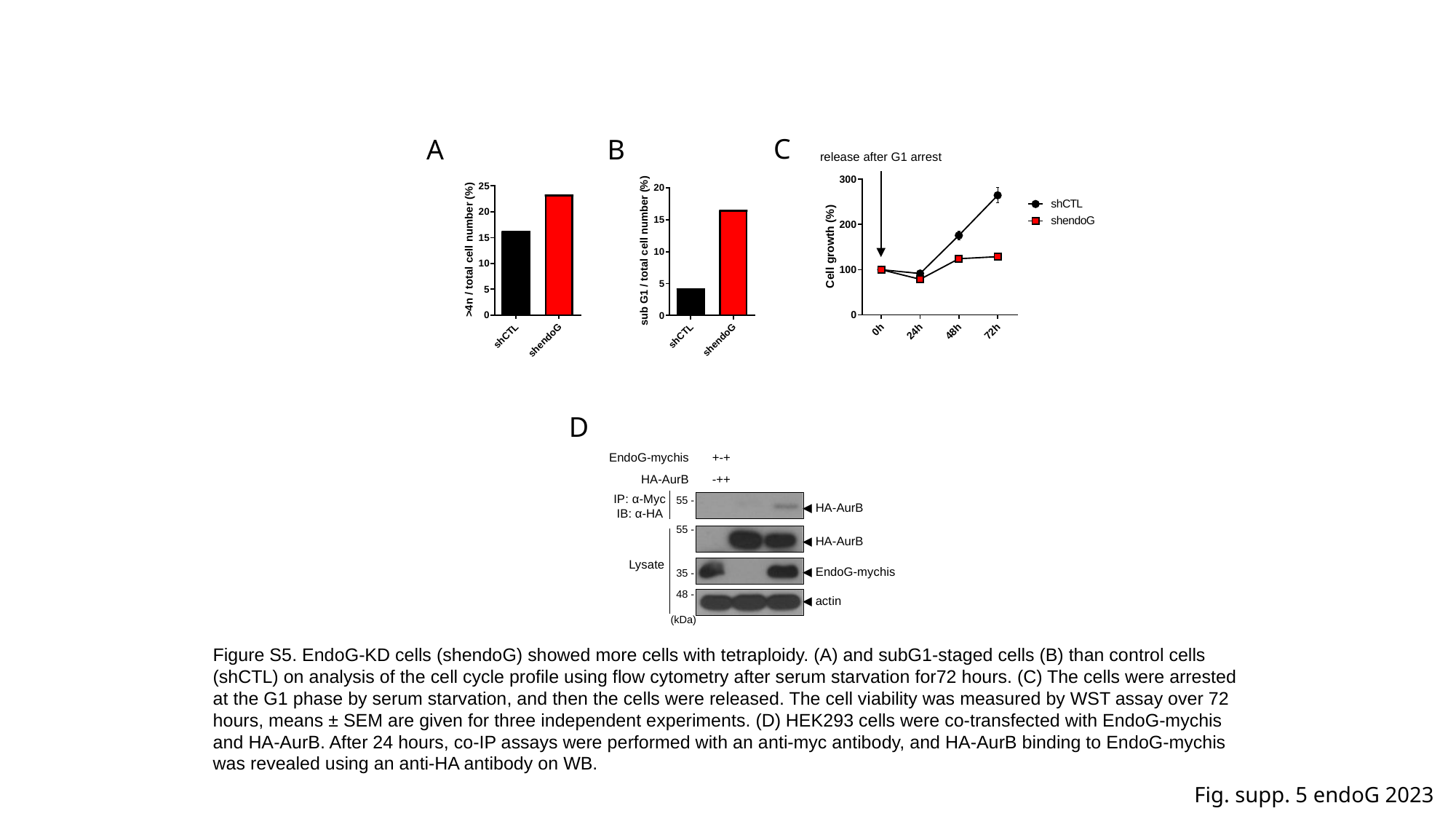

C
A
B
release after G1 arrest
D
EndoG-mychis
HA-AurB
+-+
-++
IP: α-Myc
IB: α-HA
55 -
◀ HA-AurB
55 -
◀ HA-AurB
Lysate
◀ EndoG-mychis
35 -
48 -
◀ actin
(kDa)
Figure S5. EndoG-KD cells (shendoG) showed more cells with tetraploidy. (A) and subG1-staged cells (B) than control cells (shCTL) on analysis of the cell cycle profile using flow cytometry after serum starvation for72 hours. (C) The cells were arrested at the G1 phase by serum starvation, and then the cells were released. The cell viability was measured by WST assay over 72 hours, means ± SEM are given for three independent experiments. (D) HEK293 cells were co-transfected with EndoG-mychis and HA-AurB. After 24 hours, co-IP assays were performed with an anti-myc antibody, and HA-AurB binding to EndoG-mychis was revealed using an anti-HA antibody on WB.
Fig. supp. 5 endoG 2023
